# Construction and validation of a metabolic gene-associated prognostic model for cervical carcinoma and the role on tumor microenvironment and immunity

**DOI:** 10.18632/aging.203723

**Published:** 2021-12-01

**Authors:** Jinzhi Huang, Fei Luo, Mingjie Shi, Jiaxin Luo, Choudi Ma, Shangzheng Li, Yue Wei, Runmin Guo, Ting Li

**Affiliations:** 1 Department of Obstetrics and Gynecology, Affiliated Hospital of Guangdong Medical University, Zhanjiang 524001, Guangdong, PR China; 2 Key Laboratory of Research in Maternal and Child Medicine and Birth Defects, Guangdong Medical University, Foshan 528300, Guangdong, PR China; 3 First College of Clinical Medicine, Guangdong Medical University, Zhanjiang 524001, Guangdong, PR China; 4 Department of Ultrasound, Shunde Women and Children’s Hospital (Maternity and Child Healthcare Hospital of Shunde Foshan), Guangdong Medical University, Foshan, China; 5 Department of Internal Medicine, Shunde Women and Children’s Hospital (Maternity and Child Healthcare Hospital of Shunde Foshan), Guangdong Medical University, Foshan, China; 6 Department of Pharmacy, Affiliated Hospital of Guangdong Medical University, Zhanjiang 524001, Guangdong, PR China

**Keywords:** cervical carcinoma, metabolism, prognosis, tumor microenvironment, immunity

## Abstract

Metabolic reprogramming is a common feature of tumor cells and is associated with tumorigenesis and progression. In this study, a metabolic gene-associated prognostic model (MGPM) was constructed using multiple bioinformatics analysis methods in cervical carcinoma (CC) tissues from The Cancer Genome Atlas (TCGA) database, which comprised fifteen differentially expressed metabolic genes (DEMGs). Patients were divided into a high-risk group with shorter overall survival (OS) and a low-risk group with better survival. Receiver operating characteristic (ROC) curve analysis showed that the MGPM precisely predicted the 1-, 3- and 5-year survival of CC patients. As expected, MGPM exhibited a favorable prognostic significance in the training and testing datasets of TCGA. And the clinicopathological parameters including stage, tumor (T) and metastasis (M) classifications had significant differences in low- and high-risk groups, which further demonstrated the MGPM had a favorite prognostic prediction ability. Additionally, patients with low-ESTMATEScore had a shorter OS and when those combined with high-risk scores presented a worse prognosis. Through “CIBERSORT” package and Wilcoxon rank-sum test, patients in the high-risk group with a poor prognosis showed lower levels of infiltration of T cell CD8 (*P* < 0.001), T cells memory activated (*P* = 0.010) and mast cells resting (*P* < 0.001), and higher levels of mast cells activated (*P* < 0.001), and we also found these patients had a worse response for immunosuppressive therapy. These findings demonstrate that MGPM accurately predicts survival outcomes in CC patients, which will be helpful for further optimizing immunotherapies for cancer by reprogramming its cell metabolism.

## INTRODUCTION

Cervical carcinoma (CC) is a common gynecological malignant tumor. Although both the incidence and mortality rates of CC patients have been declining in recent years due to increased screening rates for the HPV virus, it remains an important global health concern occurring across various age groups in females [1]. According to a World Health Organization (WHO) study, there were 569,847 new cases (about 3.2%) of CC were diagnosed and 311,365 patients (about 54.6%) died of this disease in 2018 alone [2]. Squamous cell carcinoma (SCC) and adenocarcinoma (AdCA) are the two most frequent histopathological subtypes of CC patients [3, 4]. Patients with early stage or localized lesions can perform surgery to achieve long-term survival, while most patients usually have advanced symptoms at the time of diagnosis. After treating with conventional therapies, such as chemotherapy, radiation therapy, or a combination of both, the survival period still cannot be extended. Therefore, there is an imminent need for improving CC screening and therapeutic methods.

It has been well known that metabolic pathways can be altered in tumor cells to acquire more nutrients for proliferation and survival [5–7]. Metabolic reprogramming could inhibit their anti-tumor activities in the immune cells, thereby influencing tumor progress and immunotherapeutic efficacy [8–10]. Studies have shown that some metabolic processes including nucleotide, vitamin/co-factor and carbohydrate were invariably related to poor prognosis. based on the mRNA expression patterns of 33 different cancers and chemoresistance of cancer cells. However, the roles of metabolic-related mechanisms and immunity in CC patients are not elucidated. The clinically feasible and superior or comprehensive models are also needed to anticipate the overall survival (OS) of tumor patients [11]. Increasingly, the fast-developing genomic profiling and the viability of big data analysis provide more convenience for determining the optimal care and evaluating prognosis of cancer patients by constructing risk models. In this study, we built a metabolic gene-associated prognostic model (MGPM) of cervical cancer based on metabolic-related genes with differentially expressed between tumor and normal tissues, assessing its correlation with tumor microenvironment and immunity to evaluate its clinical significance for the prognosis of CC. These will interpret the mechanism and the correlation of tumor immunity from the perspective of metabolism, and will all need to be further confirmed in the future.

## RESULTS

### Identified differentially expressed metabolic genes and conducted enrichment analysis

**Figure 1:**
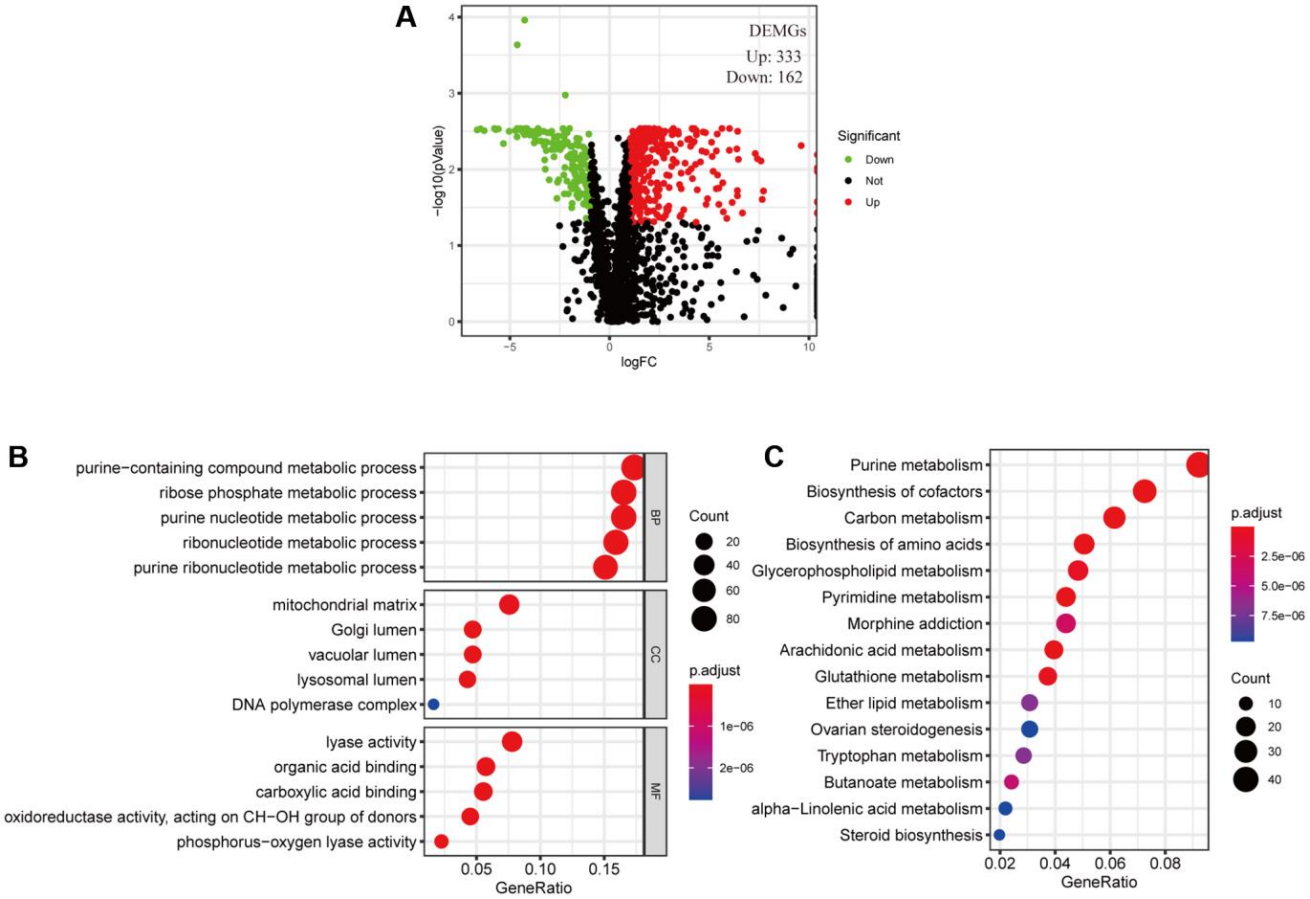
**Filtered differentially expressed metabolic genes (DEMGs) and performed Enrichment analysis.** (**A**) A volcano map presented DEMGs between the cervical cancer and the normal samples, Dots represent significantly down-regulated (Green) and up-regulated (Red) genes. (**B**) GO function annotations and (**C**) KEGG enrichment analysis.

The cancer cell metabolic gene set comprising 2071 genes was downloaded from ccmGDB (https://bioinfo.uth.edu/ccmGDB/). A total of 495 CC-specific mRNAs (1320 upregulated and 1944 downregulated) were identified as differentially expressed metabolic genes (DEMGs), with Limma package using *P* value < 0.05 and |logFC| > 1 as screening criteria. The volcano map exhibits significant differences and distribution of mRNAs in CC based on the fold change (Figure 1A). GO analysis showed that the DEMGs were significantly associated with purine-containing compound metabolic, ribose phosphate metabolic, purine nucleotide metabolic, ribonucleotide metabolic and purine ribonucleotide metabolic processes (Figure 1B). And the pathways such as purine metabolism, biosynthesis of amino acids, pyrimidine metabolism, biosynthesis of cofactors and carbon metabolism were mainly enriched in the KEGG pathways (Figure 1C).

### Construction of a metabolic prognostic model based on fifteen DEMGs for cervical carcinoma

**Figure 2:**
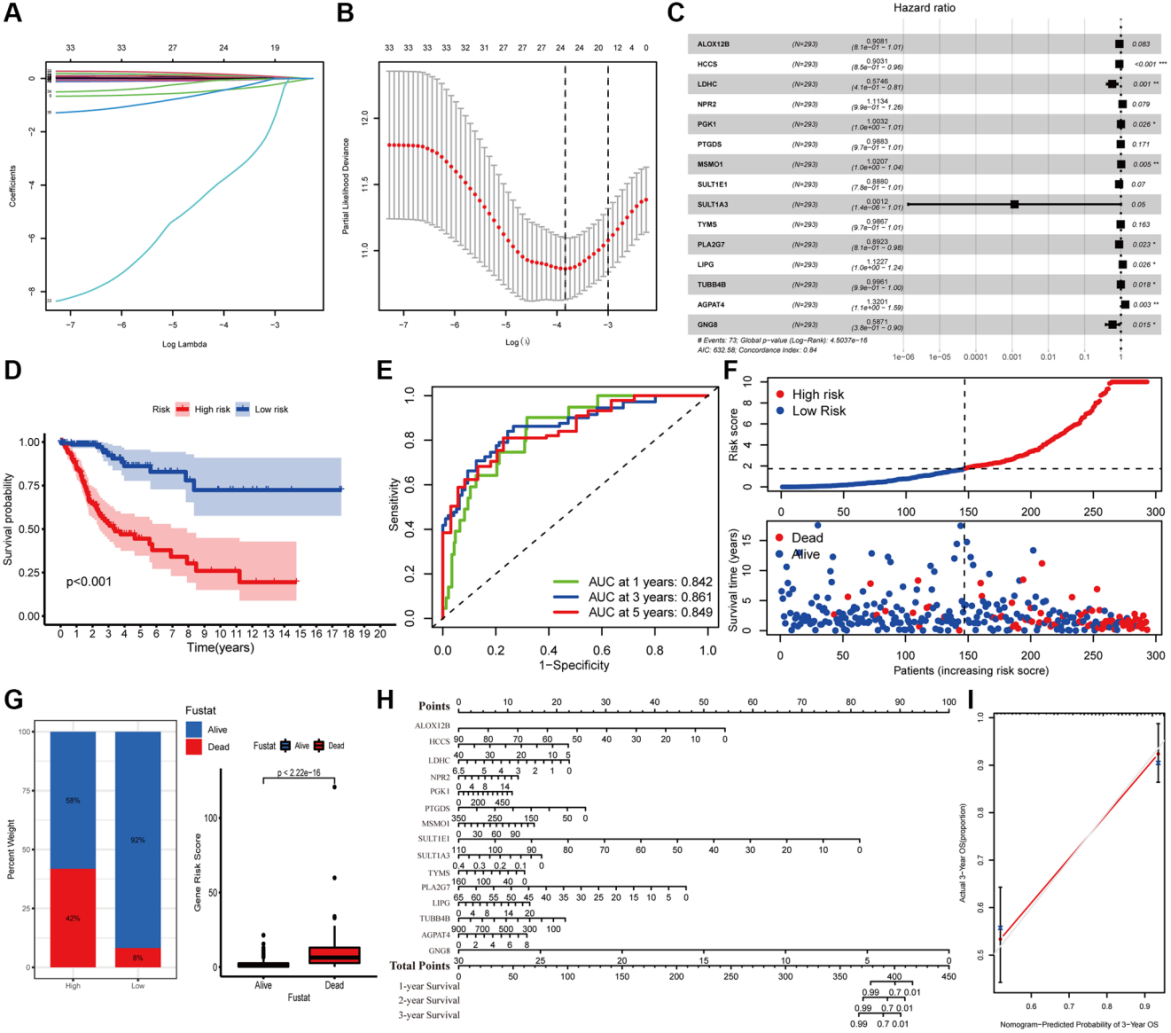
**Construction of metabolic gene-associated prognostic model (MGPM) using a series of bioinformatics technology.** (**A**, **B**) LASSO Cox regression analysis with 5-fold cross-validation and generation of coefficient outline based on the log (lambda) sequence were used to identify the potential independent prognostic risk signature genes. (**C**) Multivariate Cox regression analysis results show the *p* values and hazard ratios (HR) with confidence intervals (CI) of the fifteen DEMGs. (**D**) Kaplan-Meier survival curves show the overall survival (OS) rates of high-risk (*n* = 146) and low-risk (*n* = 147) CC patients. Patients in high-risk group had a shorter OS compared to those in low-risk group. (**E**) ROC curve analysis results show the accuracy and reliability of the MGPM in determining the 1-, 3- and 5-year survival outcomes (AUC values are shown in parentheses). (**F**) From the top to bottom, the survival status of each patient was sorted according to the low-risk (blue) and high-risk (red) scores. A scatter plot then exhibited the survival status and survival time of patients, the dots represent patients that have died (red) and alive (blue) at the time of analysis. (**G**) Analysis of the difference in survival status between the low- and high-risk groups. (**H**, **I**) The nomogram and calibration plot were employed to depict the expected survival of individual CC patients and to assess the discriminative ability of the MGPM-based risk signature, respectively.

The survival difference of the DEMGs was analyzed by Kaplan-Meier method and log-rank analysis. There are 72 genes remarkably related to the prognosis of CC (Supplementary Figure 1). A total of 15 DEMGs were incorporated into the multivariate Cox regression analysis after univariate Cox and Lasso regression analysis (Figure 2A–2C). A multivariate Cox proportional hazards regression model was subsequently utilized to build the metabolic gene-associated prognostic model (MGPM) risk signature and calculate the risk score of each individual, patients were then divided into low- and high-risk groups using the median risk score as a cut-off. And nine out of fifteen variables ((HCCS, LDHC, PGK1, MSMO1, PLA2G7, LIPG, TUBB4B, AGPAT4 and GNG8) had significant statistical difference (*P* < 0.05) in MGPM (Supplementary Table 1). The survival analysis of the MGPM using the Kaplan–Meier method indicated that the patients in the high-risk group had a shorter overall survival compared with those in the low-risk group, and there was a significant difference between them (*P* < 0.001, Figure 2D). The ROC curve analysis represented a favorite prognostic value for this model in evaluating the overall survival of patients with CC (1-year AUC = 0.842, 3-year AUC = 0.861, 5-year AUC = 0.849; Figure 2E). The survival status of each patient was evaluated according to the risk score (Figure 2F). Compared with the low-risk group, patients have a shorter survival time and a higher incidence of death in the high-risk group (Figure 2F). The percentage of deaths in the low- and high-risk groups was 42% and 8%, respectively. There is a statistical difference between them (*P* < 2.22E-16, Figure 2G). Additionally, the nomogram was employed to depict the prospective survival of CC patients (Figure 2H), and the calibration plot was performed to assess the discernment of the MGPM-based risk signatures, and the result exhibited its predicted value was virtually coincided with the actual value (Figure 2I). The clinicopathological characteristics of CC patients in this model are detailed in Supplementary Table 2.

### Validation of the MGPM-based risk signature in the training and testing cohorts

**Figure 3:**
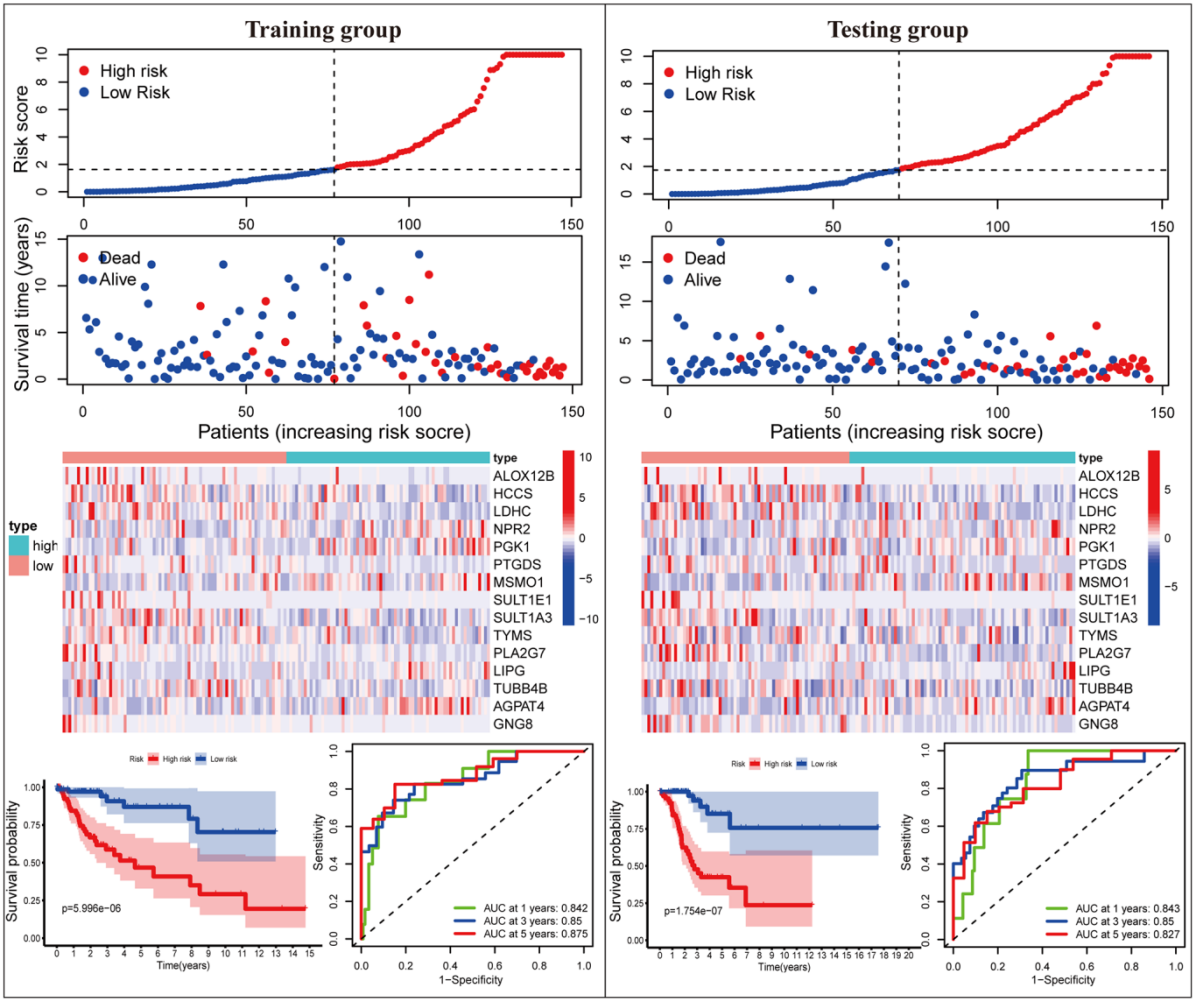
**The MGPM comprising fifteen metabolic genes was verified in the training and testing data set.** From top to bottom, the ranked dot plot illustrating the predictor-score distribution, a scatter plot presenting the patients’ overall survival status, a heatmap showing the expression profile of the fifteen signature genes of CC patients, Kaplan-Meier survival curves indicating the OS rates of high- and low-risk groups and ROC curve analysis demonstrating the accuracy and reliability of the MGPM in determining survival outcomes.

Subsequently, we proceeded with internal verification in the training (*N* = 147) and testing (*N* = 146) groups to evaluate the accuracy and robustness of the MGPM comprising fifteen DEMGs (Figure 3). From top to bottom, the risk score of each sample was calculated and ranked based on the MGPM in the training and testing data groups, respectively. And then, the overall survival status of CC patients according to the risk score distribution was presented using a scatter plot. Both data sets showed patients with high-risk score were bound up with a higher mortality rate, and the increase of risk score was proportional to the death rate in CC patients. A heatmap presenting the expression profiles of those signature genes displayed those patients in the high-risk group were more geared to elevate NPR2, PGK1, MSMO1, LIPG and AGPAT4 levels, while those in the low-risk group were more inclined to elevate ALOX12B, HCCS, LDHC, PTGDS, SULT1E1, SULT1A3, TYMS, PLA2G7, TUBB4B and GNG8 levels, these finding in the training dataset showed consist with the testing dataset. Lastly, Kaplan-Meier survival curve analysis indicated that the overall survival of patients with high-risk score was remarkably shorter than those with low-risk score in both datasets (*P* < 0.001), and ROC curve analysis also indicated there was a favorite prognostic predictive value of the MGPM both in training set (1-year AUC = 0.842, 3-year AUC = 0.861, 5-year AUC = 0.849) and testing set (1-year AUC = 0.843, 3-year AUC = 0.85, 5-year AUC = 0.827).

### Relationship between MGPM-based risk signatures and clinicopathological characteristics of CC patients

**Figure 4:**
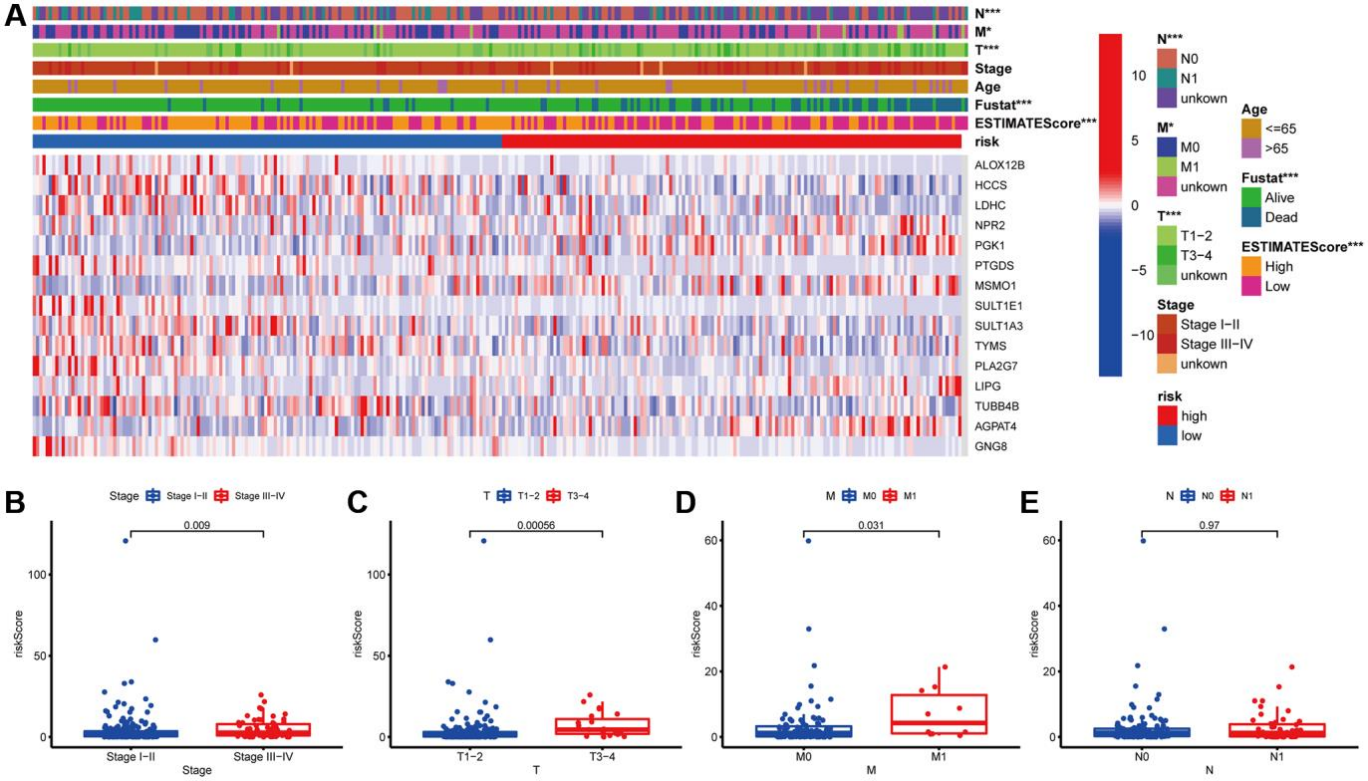
**Correlation analysis between the MGPM and clinicopathological features in low- and high-risk CC patients.** (**A**) A heatmap showed the correlation between the MGPM-based risk score and tumor microenvironment (ESTIMATEScore) or clinicopathological parameters using the Chi-square test. There were statistically significant differences in ESTIMATEScore, survival status and tumor node metastasis classification (T, N and M stages) of low- and high-risk groups. (**B**–**E**) The distribution of risk scores based on MGPM stratified according to (**B**) stage (stages I–II vs. stages III–IV), (**C**) T stage (T1-2 vs. T3-4), (**D**) M stage (M0 vs. M1) and (**E**) N stage (N0 vs. N1-2).

Next, the association between the MGPM-based risk signatures and the clinicopathological parameters was analyzed. A heatmap was employed to present the correlations between the MGPM-based risk signatures and clinicopathological variables. The results indicated that there were significant statistical differences between them regarding the tumor node metastasis classification (*P* < 0.01), survival status (*P* < 0.001) and estimate score (*P* < 0.0001) (Figure 4A). The differences in clinicopathological parameters were then evaluated between the low- and high-risk groups of MGPM by Wilcoxon rank-sum test. There are statistically significant differences in stage (*P* = 0.009) primary tumor (T, *P* = 0.00056) and lymph nodes (N, *P* = 0.031), while no statistical differences in remote metastasis (M, *P* = 0.97) (Wilcoxon signed-rank test, Figure 4B–4E).

**Figure 5:**
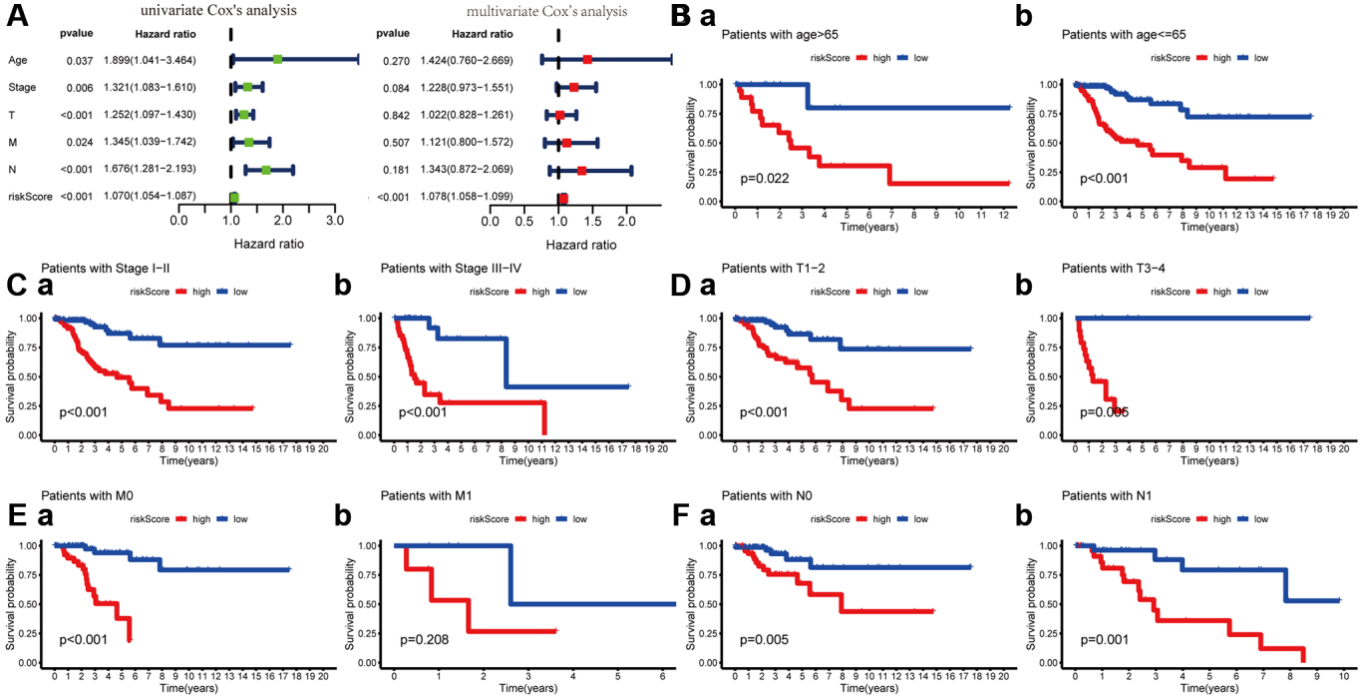
**Cox regression and Kaplan-Meier survival analysis of risk score and clinicopathological parameters.** (**A**) Cox regression analysis of risk score and clinicopathological parameters, which indicate the MPGM could as an independent prognostic factor. (**B**) Patients stratified by ages (age ≤ 65 and > 65), (**C**) Patients stratified by stage (stage I-II and stage III/IV), (**D**) Patients stratified by T stage (T1-2 and T3-4), (**E**) Patients stratified by M stage (M0 and M1) and (**F**) Patients stratified by N stage (N0 and N1).

To explore its clinical application and usability, we performed Cox regression analysis on common clinical characteristics. The results exhibited that the risk score of MGPM had statistically significant differences in both univariate- and multivariate-Cox regression groups (*P* < 0.001), which indicated the MGPM could as an independent index for prognosis to forecast the OS of CC patients (Figure 5A, Supplementary Table 3). After stratifying patients according to clinicopathological parameters, the overall survival rate of patients with the following characteristics will be significantly shortened in the high-risk group by Kaplan–Meier analysis, including age ≤ 65 (*P* < 0.001) and age > 65 (*P* = 0.022) (Figure 5B), stage I/II (*P* < 0.001) and stage III/IV (*P* < 0.001) (Figure 5C), T1-2 stage (*P* < 0.001) and T3-4 stage (*P* = 0.006) (Figure 5D), M0 stage (*P* < 0.001) (Figure 5E (a)), N0 stage (*P* = 0.006) and N1 stage (*P* = 0.001) (Figure 5F). However, there were no significance in M1 stage patients (*P* = 0.208, Figure 5E (b)) in both the high- and low-risk groups.

### The risk score based on MGPM was negatively associated with ESTIMATEScore of tumor microenvironment

**Figure 6:**
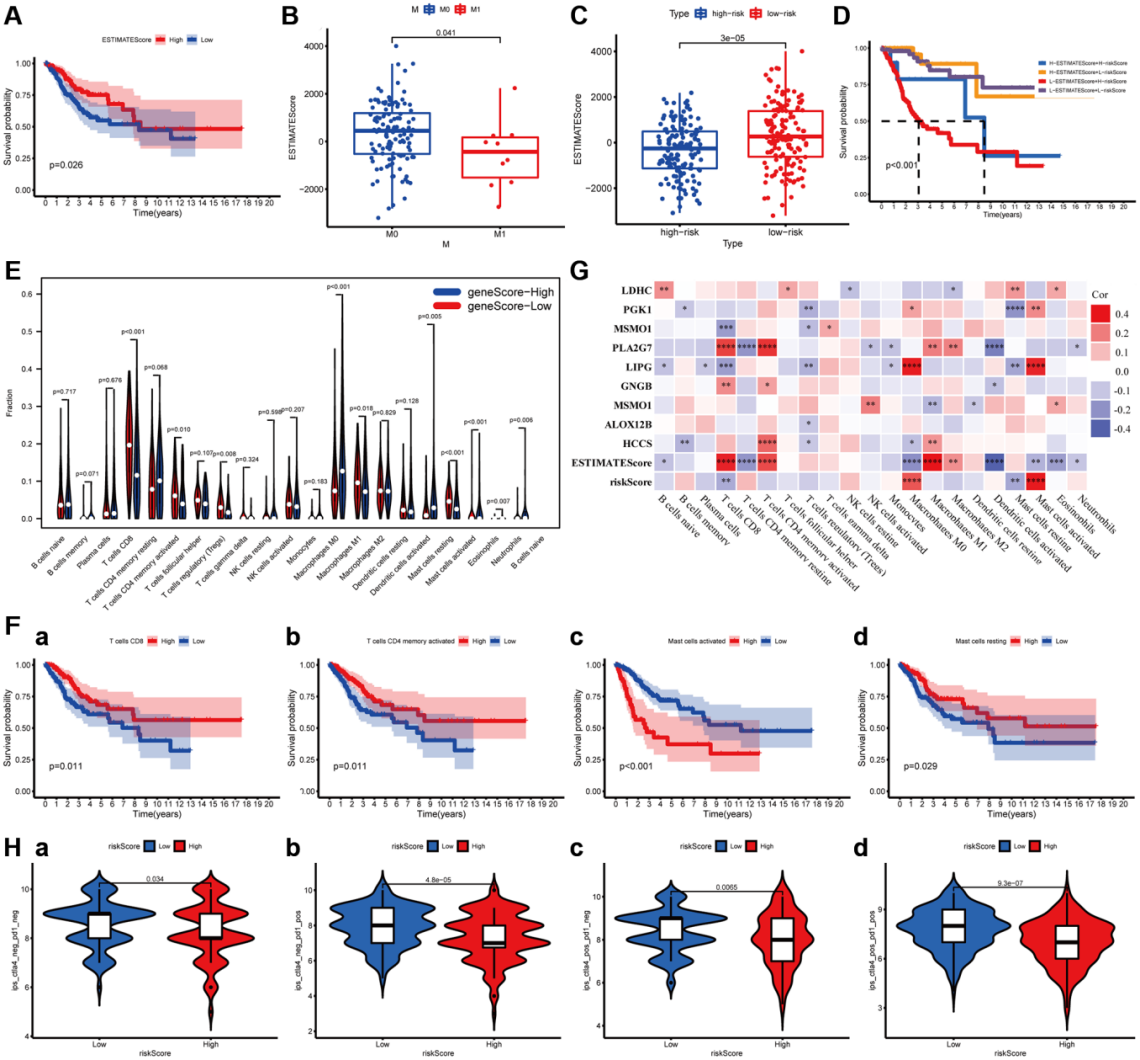
**The relationship between the MGPM-based risk score and tumor microenvironment and immune cell infiltration.** (**A**) Kaplan-Meier survival curve was utilized to evaluate the prognosis of the tumor microenvironment, the result showed low-ESTIMATEScore had a shorter OS. (**B**) The distribution of ESTIMATEScore according to clinicopathological features (M). (**C**) The difference of ESTIMATEScore in low- and high-risk groups. (**D**) Kaplan-Meier survival analysis of ESTIMATEScore in combined with a risk score. (**E**) Violin plot showed the ratio differentiation of 21 types of immune cells between CC tumor samples in low- or high-risk groups, and Wilcoxon rank-sum was applied for the significance test. (**F**) Prognosis-related immune-infiltrating cells. (**G**) The correlation analysis between 21 types of immune-infiltrating cells and risk score, ESTIMATEScore and MGPM-based risk signature genes. (**H**) Immunotherapy score of low- and high-risk groups based on TCIA database.

ImmuneScore and StromalScore have been represented scores according to the ratio of immune and stromal components in the tumor, respectively. ESTIMATEScore was considered as the summation of them, and the tumor purity of each subject was calculated based on all three scores. The overall survival of patients was positively correlated with ESTIMATEScore (*P* = 0.0026, Figure 6A), while not significantly with StromalScore and ImmuneScore (Supplementary Figure 2). Then, the data combined with the clinical information and ESTIMATEScore of CC patients were analyzed to discover the difference between them, showing that ESTIMATEScore were notably declined in M1 group compared to M0 group with a statistical significantly difference (Wilcoxon signed-rank test, *P* = 0.041, Figure 6B). These findings indicated that the components of immune and stromal were involved in the progression of CC, especially in metastasis. In the constructed MGPM, the ESTIMATEScore was higher in the low-risk group than the high-risk group with a statistically significant difference, indicating a better prognosis in CC patients (Wilcoxon signed-rank test, *P* = 3E-05, Figure 6C). In addition, the Kaplan–Meier survival curves suggested the patients with low-ESTIMATEScore and high-risk score (based on the MGPM) had a shorter survival time and a higher incidence of death (*P* < 0.001, Figure 6D).

### Relationship between the MGPM and the proportion of tumor-infiltrating immune cells

The proportion of tumor-infiltrating immune subtypes was analyzed by CIBERSORT method in CC cases (Supplementary Figure 3). Subsequently, we evaluated the important tumor-infiltrating immune cell differences in low- and high-risk groups through the Wilcoxon rank-sum test (Figure 6E). There are ten types of tumor-infiltrating immune cells with statistically significant differences, including T cells CD8 (*P* < 0.001), T cells memory activated (*P* = 0.010), T cells regulatory (Tregs) (*P* = 0.008), Macrophages M0 (*P* < 0.001), Macrophages M1 (*P* = 0.018), Dendritic cell activated (*P* = 0.005), Mast cells resting (*P* < 0.001), Mast cells activated (*P* < 0.001), Eosinophils (*P* = 0.007) and Neutrophils (*P* = 0.006). Among them, T cells CD8 (*P* = 0.011), T cells memory activated (*P* = 0.011), Mast cells resting (*P* = 0.029) and Mast cells activated (*P* < 0.001) were correlated prognosis in CC patients (Figure 6F). The co-expression analysis of twenty-one tumor-infiltrating immune cells and the MGPM-based risk signature was performed (Figure 6G). The results indicated T cells CD8 (R = −0.25, *P* = 2.37E-05), Macrophages M0 (R = 0.27, *P* = 2.24E-06), Mast cells resting (R = −0.29, *P* = 4.05E-07) and Mast cells activated (R = 0.35, *P* = 8.23E-10) were correlated with MGPM-based risk signature with statistically significant differences.

The co-expression analysis of twenty-one tumor-infiltrating immune cells and the MGPM-based risk signature and tumor microenvironment was then performed (Figure 6G). The results indicated T cell CD8 had a positive correlation with ESTIMATEScore (R = 0.34, *P* = 1.51E-09), PLA2G (R = 0.29, *P* = 2.8E-07) and GNG8 (R = 0.17, *P* = 0.003) expressions, a negative correlation with risk score of MGPM (R = −0.18, *P* = 0.002), LIPG (R = −0.21, *P* = 0.0003) and MSMO1 (R = −0.21, *P* = 0.00024) expressions. T cells regulatory (Tregs) had a positive correlation with the expressions of PGK1 (R = −0.17, *P* = 0.003), MSMO1 (R = −0.14, *P* = 0.014), LIPG (R = −0.16, *P* = 0.004), ALOX12B (R = −0.12, *P* = 0.041) and HCCS (R =−0.12, *P* = 0.045). Mast cells resting had a positive correlation with the LDHC expression (R = 0.15, *P* = 0.009), and a negative correlation with risk score (R = −0.19, *P* = 0.0012), LIPG (R = −0.19, *P* = 0.001) and PGK1 (R = −0.23, *P* = 4.25E-05) expressions. Mast cells activated had a positive correlation with risk score (R = 0.36, *P* = 1.2E−10), LIPG (R = 0.34, *P* = 2.32E-09) and PGK1 (R = 0.17, *P* = 0.003) expressions, a negative correlation with ESTIMATEScore (R = −0.16, *P* = 0.0049).

### Correlation of MGPM with the response of immune checkpoint inhibitors

To assess the relationship between MGPM and immunotherapy responses, we explored the correlation of MGPM-based risk score with common immune checkpoint inhibitors based on the TCIA database, including cytotoxic T lymphocyte antigen 4 (CTLA4) and programmed cell death 1 (PD1). The results indicated that patients in low-risk group had better responses to immunotherapy (Wilcoxon signed-rank test, *P* < 0.05, Figure 6H).

## DISCUSSION

Cervical cancer is the most common gynecological malignancy, which can be primary tumors that derived either from the genitalia or the products of conception, or secondary tumors impacted by other cancerous organs with metastasis [12]. Metabolism plays a crucial role in the process of tumor initiation, development, and recurrent [13, 14]. Metabolic reprogramming was utilized by cancer cells to achieve limited nutrient resources for proliferation, growth, survival, and long-term maintenance when competing with normal cells [15].

In this study, we identified 495 DEMGs according to transcriptome data analysis of tumor and normal cervical tissues from the TCGA database. KEGG functional enrichment analysis suggested that these DEMGs are mainly involved in pathways such as purine metabolism, biosynthesis of amino acids, pyrimidine metabolism, biosynthesis of cofactors and carbon metabolism. These pathways are all associated with metabolic-related alterations and can interfere with the development of CC patients. The data set was then analyzed through Cox- and Lasso regression analyses to identify the prognostic DEMGs and construct a MGPM. This model exhibited a favored prognosis ability, and which was verified in the training and testing data sets. Patients with high-risk score had worse overall survival compared with those with low-risk score. The ROC curve of this model suggests a favorable competence in forecasting 1-year OS (AUC = 0.842), 3-year OS (AUC = 0.861), and 5-year OS (AUC = 0.849). A nomogram was developed based on the MGPM-based risk signatures to assess the reliability and feasibility of the model and determine the best treatment strategies for CC patients. The calibration plot exhibited predicted value was virtually coincided with the actual value in MGPM. Nine out of fifteen genes (HCCS, LDHC, PGK1, MSMO1, PLA2G7, LIPG, TUBB4B, AGPAT4 and GNG8) of MGPM-based risk signature genes had statistically significant differences, meaning these genes were independent prognostic factors related to prognosis. Kaplan-Meier survival curve analysis indicated that low expression of GNG8, HCCS, LDHC, PLA2G7 and TUBB4B genes and high expression of AGPAT4, LIPG, MSMO1 and PGK1 genes were relevant to shorter survival time of CC patients.

MGPM-based risk signature genes have been reported in previous studies. Some of them play an essential role in tumor development or can independently serve as prognostic markers for tumor patients. Yet, the underlying mechanism of most of them in tumorigenesis, development and metastasis of CC patients remains unclear. For example, Lactate dehydrogenase C (LDHC) is re-expressed in sorts of tissues of malignancy, while its expression is rigidly suppressed and controlled in normal somatic tissues, making it has a highly tumor-specific [16]. Studies have shown LDHC promotes tumor cell invasion and migration by inducing epithelial-mesenchymal transition and the expression of matrix metalloproteinase-9 (MMP9), and this leads to a poor prognosis for tumor patients [17, 18]. Additionally, LDHA participates in tumor immunity by promoting upregulation of PD-L1 on tumor cells to impede effector T cell activity [19]. Moreover, serum LDHA levels are related to burthen of tumor and poor clinical outcomes to immune checkpoint (such as PD1 and CTLA4) blockade therapy [20]. Phosphoglycerate kinase 1 (PGK1) is one of the key enzymes in the Warburg effect (also known as aerobic glycolysis), and involved in energy metabolism of tumor cells [21, 22]. Many studies reported that PGK1 is highly expressed in various cancers, and its aberrant expression is associated with the poor prognosis of tumor patients [23–25]. Lipoprotein-associated phospholipase A2 (PLA2G7) silencing was recently reported that could as a novel therapy involving in anti-proliferative, anti-migratory and pro-apoptotic in tumor cells. Meanwhile, it's a novel biomarker and associated with tumor aggressiveness [26, 27]. Changes in tubulin-β4 (TUBB4B) and acylglycerophosphate acyltransferase (AGPAT) expressions have been reported with respect to tumor progression and clinic outcomes of cancer patients [28–31]. Endothelial lipase G (LIPG) is an important hydrolase involving in lipid metabolism, and it also plays a pivotal role in the tumorigenesis and development of various tumors [32–35]. Although there is currently little research data on these signature genes in cervical cancer, their aberrant expressions could be closely associated with maintaining the energy metabolism and improving the tumor microenvironment in tumor cells. Combined with our findings, metabolic differences are valuable factors in the tumorigenesis and development of CC patients, which are involved in remodeling of the immune microenvironment and cancer immunotherapy. Therefore, it’s a very feasible method and an unexplored area of research to a great extent that remodeling the tumor microenvironment and reestablishing anti-tumor immunity by targeting key players of metabolism.

Subsequently, we evaluated the effect of the risk model (MGPM) on the common clinicopathological features to authenticate its clinical feasibility. And the results indicated that the MGPM could precisely predict the clinical survival outcomes of CC patients and were notably associated with their clinicopathological traits including stage, T and M classifications. Univariate- and multivariate Cox regression analyses demonstrated that this risk model could as an independent index for prognosis in CC patients, and which clearly discriminated the patients between early- and advanced-stage through clinical symptoms. Besides, mounting evidence shows that stromal and immune components, and tumor-infiltrating immune cells have a close tie with the progress of CC [36, 37]. Another important finding of this study that there was a negative correlation between risk score originating from the MGPM and ESTIMATEScore evaluating tumor microenvironment, which could be combined to precisely predict the prognosis. Additionally, the risk score also correlated with the level of immune infiltration in CC. Among the immune infiltration cells that are differentially expressed in the high- and low-risk groups and have associated with prognosis, the MGPM-based risk score was positive correlated with the expression of T cells CD8 and mast cells resting, and negatively related to mast cells activated. The model polarized macrophages to an M1-like phenotype, and this M1 activation increased proinflammatory cytokines, promoting the infiltration and activation of CD4- and CD8 T cells in the tumor microenvironment. All these finding further confirmed that the metabolic pathways regulated by MGPM are closely related to the tumor microenvironment and immunity, making it can more accurately predict the patient's survival outcome and anti-tumor immune response effect.

Our research still has obvious deficiencies. Firstly, the construction and verification of the metabolic gene-associated prognostic model was more reliant on the data gathered and analyzed from the public databases. Thus, our findings are urgently needed to verify with more experimental and larger clinical investigations. Secondly, some specific signaling pathways on tumor growth and progress have failed to identify. Finally, we applied multiple bioinformatics methods to assess the prognostic evaluation and discriminatory ability of several MGPM-based risk signature genes in CC, while the specific functions and mechanisms of them have not been well characterized in the tumorigenesis and progression of CC patients.

Taken together, we thoroughly explored the potential biological function and prognostic ability of DEMGs in CC by a variety of bioinformatics approaches. Then, a risk model named MGPM comprising fifteen metabolic genes was constructed, nine out of them were substantiated to be independent prognostic factors that could accurately distinguish the CC patients with poor prognosis. MGPM exhibited a favorable prognostic significance and was markedly associated with the common clinicopathological parameters and immune microenvironment. These findings will be of momentous significance in revealing the etiopathogenesis of CC and developing novel and feasible targets for clinical treatment and prognostic evaluation from the perspective of metabolic reprogramming.

## MATERIALS AND METHODS

### Data selection and extraction of differentially expressed genes

RNA profiles comprising HTseq-count and fragments per kilobase per million reads (FPKM) of 306 primary CCs and 3 normal subjects were downloaded from the cancer genome atlas database (TCGA, https://portal.gdc.cancer.gov/, Supplementary Table 2). The corresponding clinical information, including general information (age, race, ect), survival outcome (survival status and days to the last follow-up) and tumor feature (stage, lymph nodes, metastases, etc) were also downloaded. After filtering the non-CC specific expression genes, the edgeR package (http://www.bioconductor.org/packages/release/bioc/html/edgeR.html) was applied to obtain differentially expressed metabolic-related genes using Padj < 0.05 and |logFC| > 1 as screening criteria.

### Function and pathway enrichment analysis of the DEMGs

A rounded enrichment analysis concerning the functions and pathways of the DEMGs was performed in CC by the clusterProfiler packages. The cellular components (CC), biological processes (BP), and molecular functions (MF), as well as the KEGG signaling pathways. GO terms and KEGG pathways were presented by the bubble chart using the *P*-value < 0.05 as a screening criterion.

### Identification of prognosis-associated metabolic genes and construction of risk model

The mRNA expression data of normal and para-carcinoma tissue samples, and with incomplete clinical information were deleted by using the Limma package. Univariate Cox and lasso regression analyses were identified the potential prognostic metabolic-related genes, and then multivariate Cox regression analysis was performed to construct metabolic gene-associated prognostic models (MGPM) and to assure that the multi-factor models were not overfitted. And the risk score of each sample was calculated through the following formula: risk score = [Coefi1] × Exp1 + [Coefi2] × Exp2 + ... [Coefin] × Expn, Coefi is the risk coefficient of each subject deduced from the LASSO-Cox mode, Exp is the expression of consensus genes. the receiver operating characteristic (ROC) curve was utilized to measure the discriminative ability of MGPM. A nomogram was formulated to present the risk score of each individual and the calibration plot was applied to evaluate the performance of the MGPM.

### CIBERSORT estimation

The CIBERSORT algorithm was utilized to evaluate the expression abundance of 22 tumor-infiltrating immune cells (TICs) in CC using *P* < 0.05 as a cut off. And then, Wilcoxon rank-sum test was executed to calculate significant differences of the TICs in the proportion between low- and high-risk groups in MGPM. Kaplan–Meier survival analysis was then applied to explore the prognosis of all TICs using *P* < 0.05 as a threshold.

### Statistical analysis

Statistical analyses were carried out by R software, with version number v3.5.2 (Package: GDCRNATools, Limma, ggplot2, rms, preprocessCore, glmnet, survminer, timeROC). All tests were two-tailed and *P* < 0.05 was supposed to have a statistical significance.

### Data availability statement

Publicly available dataset (TCGA, https://portal.gdc.cancer.gov/) was analyzed in this study.

## Supplementary Materials

Supplementary Figures

Supplementary Tables
