# Supplementary material for: Construction and validation of a metabolic gene-associated prognostic model for cervical carcinoma and the role on tumor microenvironment and immunity
Source: Aging (Albany NY). 2021 Dec 1;13(23):25072–88. doi: 10.18632/aging.203723 (PMC8714137; doi:10.18632/aging.203723)
Supplement: Supplementary Figures [file aging-13-203723-s001.pdf]

SUPPLEMENTARY FIGURES

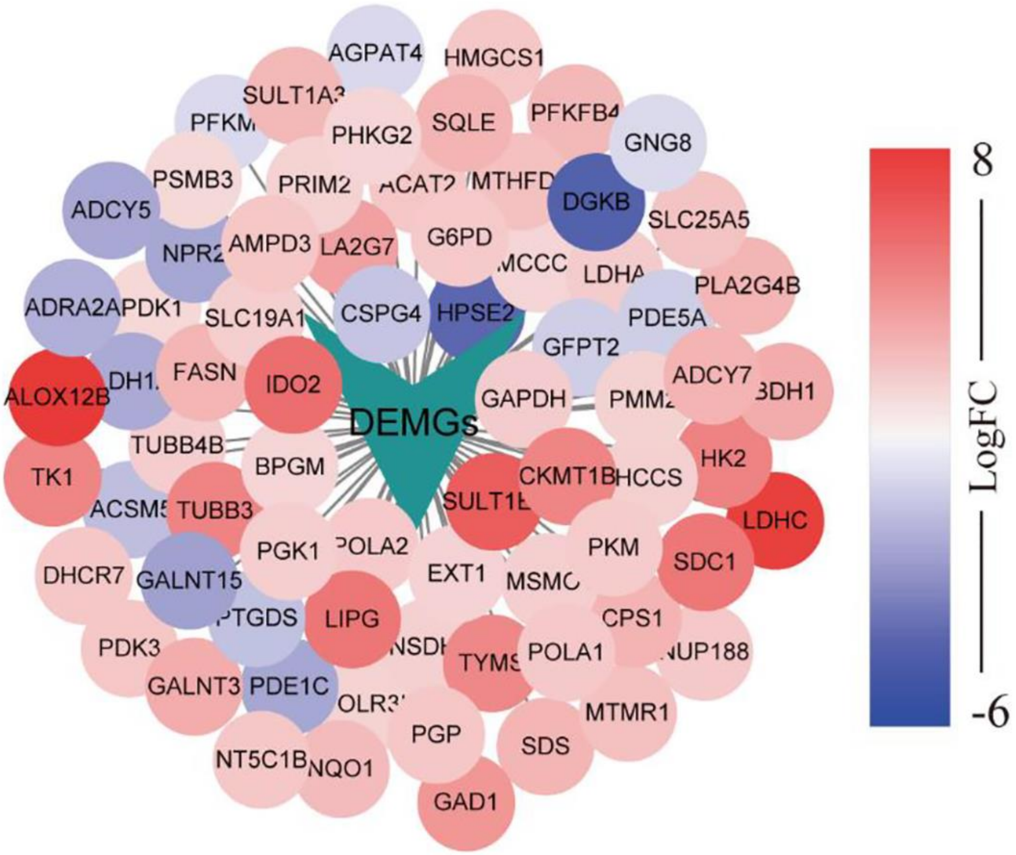

Supplementary Figure 1. 72 differentially expressed metabolic genes with prognostic ability.

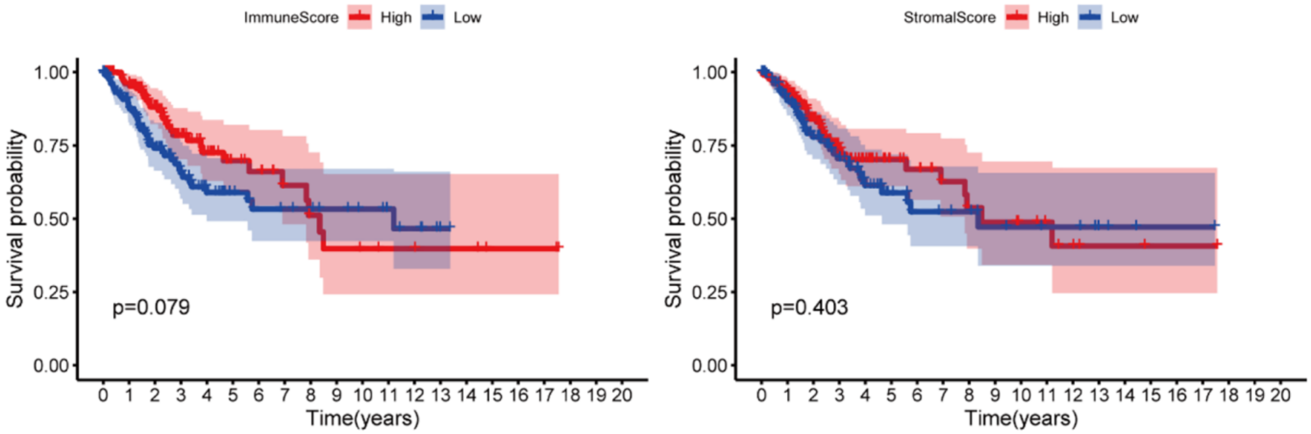

Supplementary Figure 2. Kaplan-Meier survival curve analysis of StromalScore (left) and ImmuneScore (right).

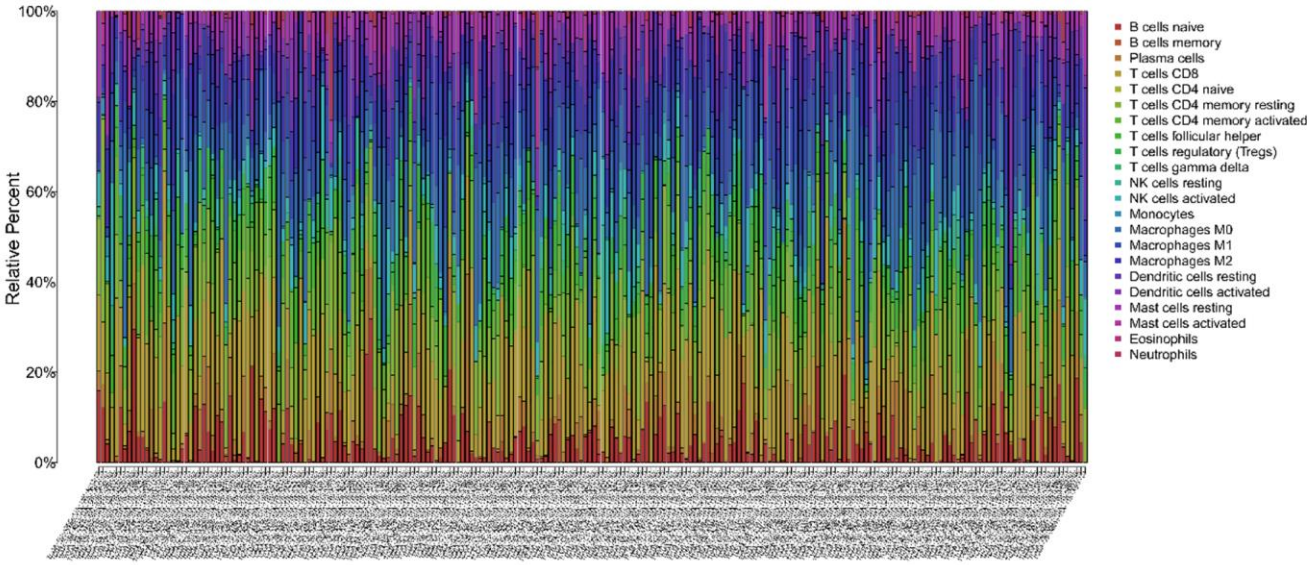

Supplementary Figure 3. Barplot shows the proportion of 21 types of tumor infiltrating cells in CC tumor samples. The column names of the plot were sample ID.
