# Supplementary material for: Construction and validation of a metabolic gene-associated prognostic model for cervical carcinoma and the role on tumor microenvironment and immunity
Source: Aging (Albany NY). 2021 Dec 1;13(23):25072–88. doi: 10.18632/aging.203723 (PMC8714137; doi:10.18632/aging.203723)
Supplement: Supplementary Tables [file aging-13-203723-s002.pdf]

SUPPLEMENTARY TABLES

Supplementary Table 1. Univariate and multivariate cox regression analysis to identify prognosis-related metabolic genes.

ID	Univariate Cox regression analysis		Multivariate Cox regression analysis		
	HR (95%CI)	*P*-value	coef	HR (95%CI)	*P*-value
AGPAT4	1.2452 (1.0732, 1.4448)	0.0038	0.2777	1.3201 (1.0961, 1.5899)	0.0034
ALOX12B	0.8876 (0.7987, 0.9864)	0.0269	−0.0964	0.9081 (0.8144, 1.0126)	0.0828
GNG8	0.5753 (0.3771, 0.8778)	0.0103	−0.5325	0.5871 (0.3823, 0.9017)	0.0150
HCCS	0.9420 (0.8981, 0.9882)	0.0144	−0.1019	0.9031 (0.8516, 0.9577)	0.0007
LDHC	0.5612 (0.3923, 0.8028)	0.0016	−0.5540	0.5746 (0.4091, 0.8071)	0.0014
LIPG	1.2120 (1.1059, 1.3282)	0.0000	0.1157	1.1227 (1.0139, 1.2431)	0.0260
MSMO1	1.0256 (1.0127, 1.0386)	0.0001	0.0205	1.0207 (1.0063, 1.0353)	0.0047
NPR2	1.1327 (1.0222, 1.2552)	0.0174	0.1074	1.1134 (0.9877, 1.2551)	0.0788
PGK1	1.0048 (1.0023, 1.0072)	0.0001	0.0032	1.0032 (1.0004, 1.0060)	0.0258
PLA2G7	0.8987 (0.8259, 0.9778)	0.0131	−0.1140	0.8923 (0.8088, 0.9844)	0.0229
PTGDS	0.9824 (0.9670, 0.9981)	0.0286	−0.0118	0.9883 (0.9717, 1.0051)	0.1705
SULT1A3	0.0002 (0.0000, 0.2583)	0.0195	−6.7515	0.0012 (0.0000, 1.0095)	0.0503
SULT1E1	0.8769 (0.7756, 0.9915)	0.0360	−0.1188	0.8880 (0.7811, 1.0096)	0.0697
TUBB4B	0.9970 (0.9945, 0.9995)	0.0190	−0.0039	0.9961 (0.9930, 0.9993)	0.0182
TYMS	0.9796 (0.9622, 0.9973)	0.0242	−0.0134	0.9867 (0.9682, 1.0055)	0.1635

Abbreviations: HR: hazard ratio; CI: confidence interval.

Supplementary Table 2. Clinicopathological characteristics statistics of CC patients.

Characteristics		No. of patients (%)	Risk group (No. of patients)	
			Low (*N* = 147)	High (*N* = 146)
**Age (years)**	≤65	260 (88.7%)	134 (91.2%)	126 (86.3%)
	>65	33 (11.3%)	13 (8.8%)	20 (13.7%)
**Stage**	I	159 (54.3%)	84 (57.1%)	75 (51.4%)
	II	64 (21.8%)	36 (24.5%)	28 (19.2%)
	III	42 (14.3%)	19 (12.9%)	23 (15.8%)
	IV	22 (7.5%)	6 (4.1%)	16 (11.0%)
	Unknown	6 (2.05%)	2 (1.4%)	4 (2.7%)
**T stage**	T1	137 (46.8%)	80 (54.4%)	57 (39.0)

	T2	68 (23.2%)	40 (27.2%)	28 (19.2%)
	T3	17 (5.8%)	5 (3.4%)	12 (8.2%)
	T4	10 (3.4%)	2 (1.4%)	8 (5.5%)
	Unknown	61 (6.1%)	20 (13.6%)	41 (28.1%)
**M stage**	M0	107 (36.5%)	66 (44.9%)	41 (28.1%)
	M1	11 (3.8%)	5 (3.4%)	6 (4.1%)
	Unknown	175 (59.7%)	66 (44.9%)	99 (67.8%)
**N stage**	N0	129 (44.0%)	78 (53.1%)	51 (34.9%)
	N1	56 (19.1%)	30 (20.4%)	26 (17.8%)
	Unknown	108 (36.9%)	39 (26.5%)	69 (47.3%)
**Survival status**	Alive	220 (75.1%)	135 (91.8%)	85 (58.2%)
	Dead	73 (24.9%)	12 (8.2%)	61 (41.8%)
**Survival time**		1072.9 ± 1145.6	1247.4 ± 1282.4	897.3 ± 957.4

Supplementary Table 3. Univariate and multivariate cox regression analysis of clinical parameters.

**ID**	**Univariate Cox regression analysis**		**Multivariate Cox regression analysis**	
	**HR (95%CI)**	***P*-value**	**HR (95%CI)**	***P*-value**
TCGA dataset (*N* = 293)				
Age	1.8986 (1.0407, 3.4639)	0.03661443	1.4242 (0.7599, 2.6692)	0.26988095
Stage	1.3209 (1.0834, 1.6109)	0.005903701	1.2284 (0.9726, 1.5514)	0.084245145
T	1.2524 (1.0968, 1.4300)	0.00088526	1.0215 (0.8279, 1.2605)	0.842492879
M	1.3454 (1.0393, 1.7417)	0.024323173	1.1211 (0.7997, 1.5717)	0.507094822
N	1.6765 (1.2814, 2.1934)	0.000164465	1.3432 (0.8721, 2.0688)	0.180549318
riskScore	1.0704 (1.0539, 1.0871)	7.85E-18	1.0785 (1.0584, 1.0990)	3.62E-15
